# Role of TRP channels in lymphatic function

**DOI:** 10.3389/fphys.2026.1883288

**Published:** 2026-07-23

**Authors:** Xinyu Cao, Yen-Lin Chen

**Affiliations:** Department of Pharmacology, Toxicology & Neuroscience, Louisiana State University Health Sciences-Shreveport, Shreveport, LA, United States

**Keywords:** Ca^2+^ signaling, contraction, lymph transport, pathological, TRP channels

## Abstract

Accumulating evidence establishes intracellular Ca^2+^ signaling as a central regulator of lymphatic endothelial function, with transient receptor potential (TRP) channels emerging as key molecular regulators that may translate mechanical, chemical, and inflammatory cues into lymphatic vascular responses. TRP channel–mediated signaling influences multiple aspects of lymphatic biology, including vessel development, endothelial barrier regulation, contractility, and lymph transport. Dysregulation of these pathways contributes to lymphatic dysfunction in inflammatory, metabolic, and cardiovascular diseases. A deeper mechanistic understanding of TRP channel signaling in the lymphatic vasculature may reveal novel therapeutic targets for lymphatic disorders and related pathologies. This review summarizes current evidence on the roles of TRP channels in the lymphatic vascular system.

## 1 Introduction

The lymphatic system plays a central role in maintaining tissue fluid homeostasis, waste clearance, immune surveillance, and lipid transport (Oliver et al., 2020), functions distinct from those of the blood vasculature. These functions depend on efficient lymph propulsion, which is tightly regulated by intracellular Ca^2+^ signaling. In collecting lymphatic vessels, rhythmic contractions generated by lymphatic muscle cells drive active lymph pumping, a process that requires precisely coordinated Ca^2+^ dynamics. Ca^2+^ influx through L-type voltage-gated calcium channels has been shown to contribute critically to lymphatic pacemaking activity and spontaneous contractions. In addition to intrinsic contractile mechanisms, extrinsic forces—including skeletal muscle movement, arterial pulsation, and respiration—further support lymph flow (Zweifach and Prather, 1975; Engeset et al., 1977; Olszewski and Engeset, 1980; Lee et al., 2014; Scallan et al., 2016). Beyond voltage-gated calcium channels, accumulating evidence suggests that additional Ca^2+^-permeable pathways contribute to lymphatic function. In particular, Transient Receptor Potential (TRP) channels, non-selective cation channels, have emerged as important regulators of intracellular Ca^2+^ signaling across multiple vascular beds and transduce diverse physical and chemical stimuli, including mechanical stress, temperature changes, and ligand binding, into intracellular Ca^2+^ signals.

**Figure 1:**
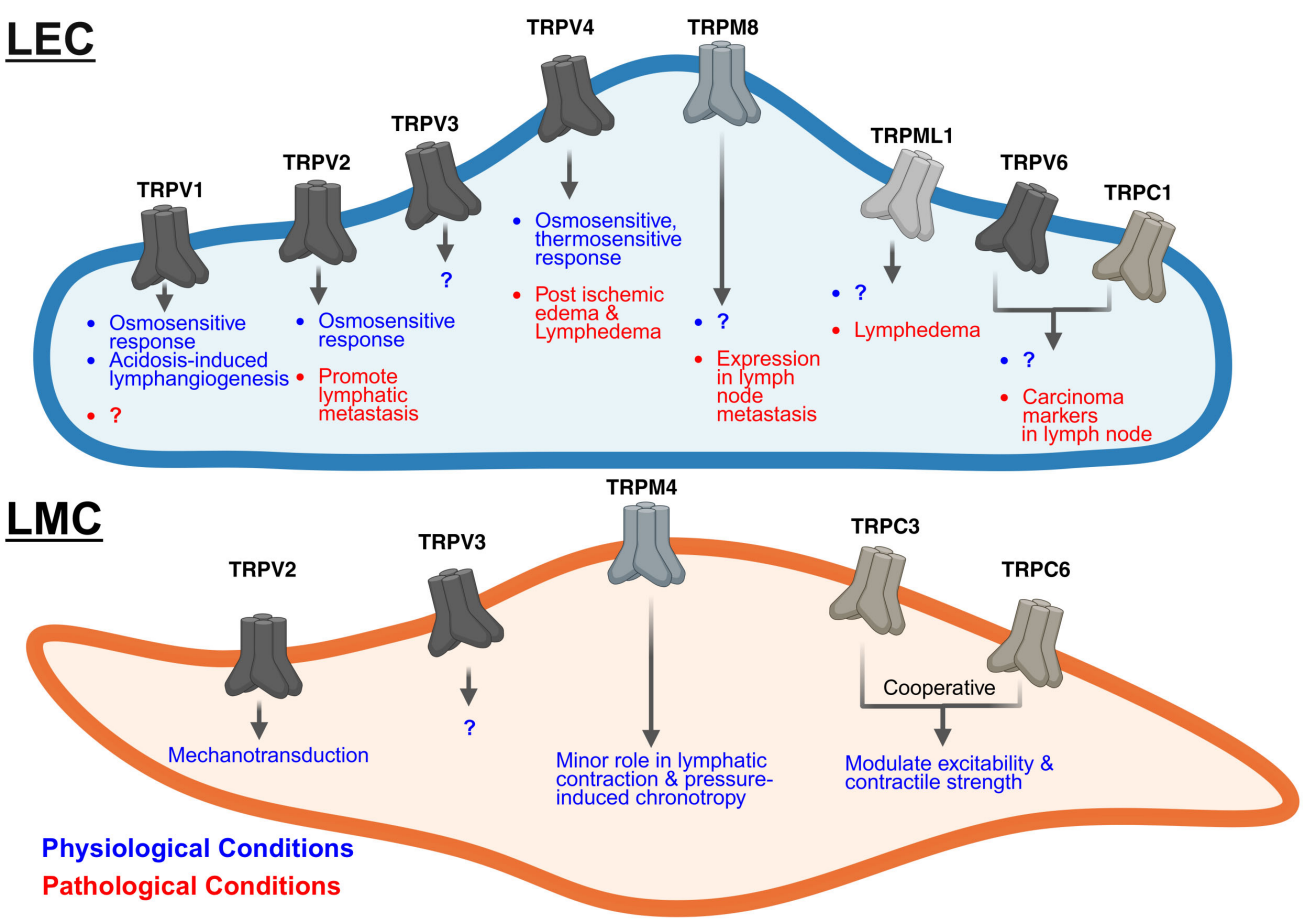
Schematic summarizing current findings on lymphatic TRP channels in physiological (Blue) and pathological (Red) conditions (LEC: lymphatic endothelial cell; LMC: lymphatic muscle cell; question mark: no current research reported; modified using BioRender).

The mammalian TRP channel superfamily comprises several major subfamilies—TRPV (Transient Receptor Potential Vanilloid), TRPA (Transient Receptor Potential Ankyrin), TRPC (Transient Receptor Potential Canonical), TRPM (Transient Receptor Potential Melastatin), TRPML (Transient Receptor Potential Mucolipin), and TRPP (Transient Receptor Potential Polycystic) — many of which are broadly expressed and permeable to Ca^2+^ and other cations (Cox et al., 2024). For a general overview of the TRP channel superfamily, most TRPV channels (TRPV1–4) were initially characterized as thermosensitive (Samanta et al., 2018; Zhang et al., 2023). TRPA1 is the sole TRPA channel expressed in humans and functions as a polymodal nociceptor that detects diverse noxious stimuli, including extreme cold, irritant compounds, and mechanical stress (Samanta et al., 2018; Zhang et al., 2023). TRPC channels, like most members of the TRP family, operate as Ca^2+^-permeable plasma membrane channels with variable Ca^2+^ selectivity (Vazquez et al., 2004). TRPM channels are implicated in a broad range of physiological and pathological processes, such as thermosensation, (Garcia-Avila and Islas, 2019) vascular development, (Smani et al., 2018) endothelial dysfunction, (Gatica et al., 2020) and inflammation (Zierler et al., 2017). TRPML channels primarily regulate intracellular trafficking processes, such as organelle acidification, vesicle fusion, and endosomal maturation (Samanta et al., 2018; Zhang et al., 2023). TRPP channels comprise integral membrane proteins, mutations of which are associated with autosomal dominant polycystic kidney disease (ADPKD) (Samanta et al., 2018; Zhang et al., 2023). While some select TRP channels have been studied in blood vascular endothelium and smooth muscle, their roles in the lymphatic vasculature remain incompletely understood. Given the strong dependence of lymphatic contractility and function on Ca^2+^ signaling, elucidating TRP channel contributions to lymphatic biology is particularly important. In this review, we summarize and discuss current knowledge of TRP channel expressions and functions in the lymphatic vascular system under physiological conditions and in disease (**Figure 1**) (**Table 1**), highlighting key gaps and emerging directions for future investigation.

**Table 1:** Functions of lymphatic TRP channels in physiological conditions and diseases.

Channel	Conductance and ionic selectivity	Physiological conditions	Diseases
TRPV1	• Conductance ≈ 40–100 pS • Ca^2+^ > Mg^2+^> K^+^ ≈ Na^+^ (Caterina et al., 1997; Lukacs et al., 2013)	Sensory mediator for acidosis-induced response (Nakanishi et al., 2016) and osmolarity (Solari et al., 2023)	N/A
TRPV2	• Conductance ≈ 28–100 pS • Ca^2+^ > Mg^2+^> K^+^ ≈ Na^+^ (Peralvarez-Marin et al., 2013; Zhang et al., 2016)	Mechanotransduction (Nilius and Droogmans, 2001; Davis et al., 2025)	Facilitate AKT/mTOR−dependent autophagy (Wang et al., 2023)
TRPV3	• Conductance ≈150–200 pS • Ca^2+^ > K^+^ ≈ Na^+^ (Xu et al., 2002)	Gene expression reported (Solari et al., 2020) but functions remain unclear	N/A
TRPV4	• Conductance ≈ 90 pS • Ca^2+^ > Mg^2+^> K^+^ > Na^+^ (Clapham et al., 2003; Sanchez-Hernandez et al., 2024)	Mechanosensory mediator, flow-dependent, osmolarity, and thermosensory regulator (Behringer et al., 2017; Solari et al., 2020, Solari et al., 2023; DuToit et al., 2024; Schulz et al., 2025)	Contributor to post−ischemic brain edema and lymphedema (Li et al., 2025; Schulz et al., 2025)
TRPM4	• Conductance ≈ 23.8 pS • Na^+^, K^+^ (Hof et al., 2016)	Minor contributor to lymphatic contraction and pressure−induced chronotropy (Davis et al., 2025)	N/A
TRPM8	• Conductance ≈ 83 pS • Ca^2+^ ≈ Na^+^ ≈ K^+^ (McKemy et al., 2002)	Protein and gene expression in human reactive lymphoid tissues (Hirai et al., 2018)	Lymph node metastases exhibit TRPM8 channel (Lunardi et al., 2021)
TRPML1	• Conductance ≈ 45–76 pS • Ca^2+^ > Na^+^ ≈ K^+^ (Xu et al., 2007; Zhang et al., 2012; Schmiege et al., 2018)	N/A	Precipitating factor in the pathogenesis of lymphedema (Yang et al., 2024)
TRPV6/ TRPC1	TRPV6 • Conductance ≈ 42 pS • Ca^2+^ (Yue et al., 2001) TRPC1 • Conductance ≈ 17 pS • Ca^2+^ ≈ Na^+^ ≈ K^+^ (Skopin et al., 2013; Bacsa et al., 2020)	N/A	Prognostic marker in lymph node–positive with carcinomas (Bodding et al., 2003; Azimi et al., 2017)
TRPC3/ TRPC6	TRPC3 • Conductance ≈ 42–66 pS • Ca^2+^ ≈ Na^+^ ≈ K^+^ (Wang and Zheng, 2011; Bacsa et al., 2020) TRPC6 • Conductance ≈ 15–45 pS • Ca^2+^ > Na^+^ ≈ K^+^ (Dietrich and Gudermann, 2007; Shi et al., 2010)	Cooperatively modulate lymphatic muscle excitability and contractile strength (Davis et al., 2025)	N/A

*N/A means there is no current research reported.

## 2 Lymphatic TRP channels in physiological conditions

### 2.1 TRPV1

Nakanishi and colleagues investigated the effects of acidic microenvironments on lymphatic endothelial cells (LECs) and their contribution to lymphangiogenesis (Nakanishi et al., 2016). Although TRPV1 (Transient receptor potential vanilloid 1) expression has been reported to be relatively low in rat diaphragmatic lymphatic vessels (Solari et al., 2020), TRPV1 has been identified as a key mediator of acidosis−induced responses in primary human adult dermal LECs. Specifically, acidic stimulation promoted LEC proliferation by inducing TRPV1-dependent IL−8 signaling (Nakanishi et al., 2016). These findings suggest a mechanistic link between extracellular pH sensing and lymphatic endothelial function (Nakanishi et al., 2016). However, the available evidence is derived primarily from *in vitro* cell culture models. As a result, the physiological relevance and *in vivo* roles of TRPV1 signaling in LEC biology and lymphangiogenesis remain to be fully elucidated (Nakanishi et al., 2016). In addition, Solari and colleagues demonstrated that microenvironmental osmolarity modulates spontaneous lymphatic contraction frequency, thereby influencing lymph formation and propulsion. TRPV1 expressed in lymphatic vessels may contribute to the molecular mechanisms underlying this osmosensitive response (Solari et al., 2023).

### 2.2 TRPV2

The TRPV2 channel is an osmosensitive, nonselective cation channel implicated in mechanosensory signaling (Nilius and Droogmans, 2001). Recent work by Davis and colleagues reported high TRPV2 expression in lymphatic muscle cells (LMCs) from mouse popliteal lymphatic vessels, highlighting TRPV2 as a potential contributor to lymphatic mechanotransduction. Pharmacological evidence further suggests that TRPV2 channels preferentially modulates lymphatic vessel contractility, with a more limited role in pressure−induced chronotropic regulation (Davis et al., 2025).

### 2.3 TRPV3

Relatively low TRPV3 gene expression has been reported in rat diaphragmatic lymphatic vessels (Solari et al., 2020). However, the physiological significance of TRPV3 channel signaling in lymphatic vessels remains unclear. Further studies are required to define the functional relevance of TRPV3 channel and to address this gap in our understanding of lymphatic vascular biology.

### 2.4 TRPV4

Accumulating evidence supports a central role for intracellular Ca^2+^ signaling in LECs as a regulator of collecting lymphatic vessel function, with the mechanosensitive and TRPV4 channel emerging as a key contributor. Early work by Behringer and colleagues demonstrated dynamic Ca^2+^ signaling in LECs from mouse popliteal collecting lymphatic vessels and identified TRPV4 as an important mediator of Ca^2+^ entry. Activation of the lymphatic endothelium with acetylcholine or pharmacological TRPV4 agonists elevated intracellular Ca^2+^ levels and promoted Ca^2+^ and Na^+^ influx through TRPV4 channels, leading to robust endothelial depolarization. Given the lack of an endothelium-derived hyperpolarization (EDH) pathway in lymphatic endothelium, an induced electrical response has been proposed to facilitate the rapid longitudinal spread of contraction waves along lymphatic vessels, thereby promoting coordinated lymph propulsion (Behringer et al., 2017).

Subsequent studies have extended the functional relevance of TRPV4 signaling to the flow−dependent regulation of lymphatic pumping. DuToit and colleagues showed that increased luminal flow reduces contraction frequency in rat mesenteric collecting lymphatic vessels, an adaptive response dependent on TRPV4 activation in LECs, where TRPV4-mediated Ca^2+^ influx is proposed to activate endothelial nitric oxide synthase (eNOS) and cyclooxygenase (COX) signaling, thereby coupling shear stress sensing to the endothelial-dependent release of vasoactive mediators that modulate lymphatic contractility (DuToit et al., 2024). Flow−induced mechanotransduction is well established for the mechanosensitive channel Piezo1, which serves as a primary flow sensor, and Piezo1 has been proposed to function upstream of TRPV4 signaling in non−lymphatic cells (Swain et al., 2020; Morozumi et al., 2021; Swain and Liddle, 2021). Whether Piezo1−dependent mechanotransduction similarly engages TRPV4 signaling in LECs remains unclear and requires further investigation. In addition to mechanical sensing, lymphatic TRPV4 channel has also been implicated in thermosensory and osmolarity induction. Environmental temperature (Solari et al., 2020) or osmolarity (Solari et al., 2023) can activate TRPV4 channels in diaphragmatic lymphatic vessels, altering contraction frequency and lymph flow. These findings positioned TRPV4 as a sensor that links ambient thermal and osmolar changes to lymphatic pumping behavior, highlighting the channel’s responsiveness to diverse physiological stimuli.

Transcriptomic analyses further reinforce the importance of TRPV4 in lymphatic regulation. Single−cell RNA−sequencing studies of mouse collecting lymphatic vessels revealed high TRPV4 gene expression in LECs and in a subset of Lyve1-postive macrophages, whereas LMCs exhibit relatively low expression. Consistent with a role for intercellular signaling, Schulz and colleagues reported that pharmacological activation of TRPV4 leads to contractile dysregulation in isolated collecting lymphatic vessels (Schulz et al., 2025). These effects were proposed to arise indirectly through paracrine mechanisms, including prostanoid−mediated activation of thromboxane A_2_ receptors (TXA_2_Rs) in LMCs by TRPV4-positive myeloid cells, as well as increased nitric oxide production by LECs.

### 2.5 TRPM4

Although TRPM4 gene is detectable in a subset of lymphatic muscle cells by single−cell transcriptomic analysis, functional studies suggest that TRPM4 plays a minimal role in lymphatic contraction and pressure−induced chronotropy (Davis et al., 2025).

### 2.6 TRPM8

TRPM8 channel protein and gene expression have been identified in human reactive lymphoid tissues and mature B cell neoplasms, with predominant expression in pre-plasmablasts, plasmablasts, plasma cells, and mature B cell lymphomas exhibiting plasmacytic differentiation (Hirai et al., 2018). But the physiological role of TRPM8 channel in lymphatic vasculature remains poorly defined, underscoring a critical need for further investigation.

### 2.7 TRPC3/TRPC6

TRPC3 channel and TRPC6 channel are diacylglycerol−activated, non−selective cation channels that share similar activation mechanisms and biophysical properties. Early genetic studies demonstrated that global deletion of TRPC6 channels results in compensatory upregulation of TRPC3 channel, supporting the concept of functional redundancy within the TRPC family (Dietrich et al., 2005). In LMCs, however, TRPC3 gene expression is detected at very low levels in only a small subset of cells and does not appear to contribute to popliteal lymphatic vessel contractile patterns or pressure−induced lymphatic chronotropy (Davis et al., 2025). Although TRPC6 expression is modestly higher than that of TRPC3, it similarly shows no clear association with lymphatic contractile function or with pressure−dependent changes in contraction frequency (Davis et al., 2025). In contrast to these murine findings, TRPC6 protein expression has been observed in lymphatic vessels within human lymph nodes, suggesting conserved expression of this channel in lymphatic vasculature across species (Daum et al., 2024).

Notably, combined deletion of TRPC3 and TRPC6 in LMCs from popliteal lymphatic vessels produces a complex phenotype characterized by reduced contraction amplitudes across multiple transmural pressures, accompanied by increased contraction frequency and elevated basal tone, without altering pressure−induced chronotropy (Davis et al., 2025). These findings indicate that, despite their limited individual contributions, TRPC3 channels and TRPC6 channels together modulate lymphatic muscle excitability and contractile strength rather than serving as primary mediators of lymphatic pressure sensing. Collectively, available evidence suggests that TRPC3 channel and TRPC6 channel−dependent signaling may play context−dependent or cooperative roles in lymphatic vessel function. However, the physiological significance of these channels in lymphatic vascular biology remains poorly defined, highlighting a need for further investigation using cell−specific or disease−relevant models.

## 3 Lymphatic TRP channels in diseases

### 3.1 TRPV2

Wang and colleagues showed that TRPV2 channels facilitate AKT/mTOR−dependent autophagy, thereby promoting proliferation, migration, and invasion of head and neck squamous cell carcinoma cells and ultimately contributing to lymphatic metastasis (Wang et al., 2023). However, the role of TRPV2 channels in lymphatic vessels under disease conditions remains poorly understood and warrants further investigation.

### 3.2 TRPV4

Li and colleagues demonstrated that pharmacological inhibition of TRPV4 attenuates glymphatic system dysfunction and reduces cerebral edema following ischemic stroke (Li et al., 2025), implicating TRPV4 activation as a contributor to post−ischemic brain edema. Supporting a broader role for TRPV4 in lymphatic−associated inflammatory responses, a clinical study reported that breast cancer–related lymphedema is associated with increased infiltration of macrophages coexpressing lymphatic vessel endothelial hyaluronan receptor 1 (LYVE1) and TRPV4 (Schulz et al., 2025).

### 3.3 TRPML1

Yang and colleagues provided compelling evidence that TRPML1 in human LECs functionally interacts with aquaporin-3 (AQP3) and aquaporin-5 (AQP5). Using global TRPML1 knockout mouse models, they showed that TRPML1 regulates the membrane localization of AQP3 and AQP5, thereby enhancing LEC permeability and impairing lymphatic transport. These alterations promote inflammatory and fibrotic remodeling characteristic of lymphedema. Collectively, their findings position TRPML1 as a critical regulator and potential precipitating factor in the pathogenesis of lymphedema (Yang et al., 2024).

### 3.4 TRPV6/TRPC1/TRPM8

Bodding and colleagues demonstrated that the human lymph node–derived prostate cancer cell line LNCaP expresses TRPV6 gene and functional TRPV6 channels; however, TRPV6 does not contribute to store−operated Ca^2+^ entry in these cells (Bodding et al., 2003). In contrast, TRPC1 channel facilitates breast cancer cell adaptation to hypoxic stress and promotes a more aggressive phenotype. TRPC1 supports activation of multiple oncogenic signaling pathways, including Akt–HIF−1α, EGFR, STAT3, autophagy, and epithelial–mesenchymal transition (EMT). Elevated TRPC1 gene expression is associated with increased invasiveness, metastasis, and poor clinical outcomes and serves as a prognostic marker in basal breast cancer, particularly in lymph node–positive disease (Azimi et al., 2017). In malignant prostate lesions, TRPM8 channel protein expression is frequently elevated in advanced prostate cancer stages. Notably, lymph node metastases exhibit comparable TRPM8 channel protein levels to those observed in matched primary tumors (Lunardi et al., 2021). Despite these established roles in carcinoma, the functions of TRPV6, TRPC1, or TRPM8 in lymphatic endothelial cells within lymph nodes under pathological conditions, including cancer, remain largely undefined and warrant further investigation.

## 4 Conclusion

Overall, although limited, current evidence supports the view that a subset of TRP channels functions as key integrators governing lymphatic pumping, barrier integrity, and immune interactions. Dysregulation of certain channels has been associated with lymphatic dysfunction in conditions such as lymphedema, chronic inflammation, obesity, and tumor metastasis, underscoring their potential clinical relevance.

Beyond TRPV4, most TRP channels remain poorly defined in lymphatic vessels despite compelling functional parallels in blood endothelium. Channels such as TRPA1 (Bodkin and Brain, 2011; Sullivan et al., 2015), TRPM7 (Zeng et al., 2015; Wu et al., 2023), TRPML1 (Scotto Rosato et al., 2019), and TRPP (AbouAlaiwi et al., 2009) are poised to regulate lymphatic permeability, mechanotransduction, and cellular homeostasis, yet direct evidence is limited. Addressing this gap—particularly in lymphatic muscle cells—will require refined genetic and pharmacological tools to elucidate the functions of lymphatic TRP channels and to assess their viability as therapeutic targets in diseases characterized by impaired lymph transport.
